# Equivalence and practice effect of alternate forms for Malay version of Auditory Verbal Learning Test (MAVLT)

**DOI:** 10.17179/excli2015-280

**Published:** 2015-07-07

**Authors:** Norulsuhada Munjir, Zahiruddin Othman, Rahimah Zakaria, Nazlahshaniza Shafin, Noor Aini Hussain, Anisah Mat Desa, Asma Hayati Ahmad

**Affiliations:** 1 Department of Psychiatry and Mental Health, Hospital Tengku Ampuan Afzan, 25100 Kuantan, Pahang, Malaysia; 2 Department of Psychiatry, School of Medical Sciences, Universiti Sains Malaysia, 16150 Kubang Kerian, Malaysia; 3 Department of Physiology, School of Medical Sciences, Universiti Sains Malaysia, 16150 Kubang Kerian, Malaysia; 4 Nursing Program, School of Health Sciences, Universiti Sains Malaysia, 16150 Kubang Kerian, Malaysia

**Keywords:** MAVLT, alternate forms, practice effect, equivalency, neuropsychological testing

## Abstract

This study aims to develop two alternate forms for Malay version of Auditory Verbal Learning Test (MAVLT) and to determine their equivalency and practice effect. Ninety healthy volunteers were subjected to the following neuropsychological tests at baseline, and at one month interval according to their assigned group; group 1 (MAVLT - MAVLT), group 2 (MAVLT – Alternate Form 1 - Alternate Form 1), and group 3 (MAVLT - Alternate Form 2 - Alternate Form 2). There were no significant difference in the mean score of all the trials at baseline among the three groups, and most of the mean score of trials between MAVLT and Alternate Form 1, and between MAVLT and Alternate Form 2. There was significant improvement in the mean score of each trial when the same form was used repeatedly at the interval of one month. However, there was no significant improvement in the mean score of each trial when the Alternate Form 2 was used during repeated neuropsychological testing. The MAVLT is a reliable instrument for repeated neuropsychological testing as long as alternate forms are used. The Alternate Form 2 showed better equivalency to MAVLT and less practice effects.

## Abbreviations

AVLT, auditory verbal learning test; MAVLT, Malay version of auditory verbal learning test; HUSM, Hospital Universiti Sains Malaysia; AF 1, Alternate Form 1; AF 2, Alternate Form 2

## Introduction

The neuropsychological test for evaluation of memory is used widely in outpatients, particularly in the elderly populations, as well as in research settings. While re-administering the tools is necessary to assess the progression (improvement or worsening) of an illness and treatment purpose, the degree to which the result is influenced by practice effect is a matter of ongoing concern. The issue of repeatedly administering the same test also applies in WHO/UCLA auditory verbal learning test (AVLT). Although this tool has been validated and found to be a good measure, the effect of repeating the same tools is producing potential errors in measurement. 

Particularly in a memory test, participants may improve their scores when given similar test and procedure after a certain interval, which may be due to better test-taking strategies. This is referred as test-specific practice effect. Multiple strategies have been used to reduce this error including the use of match-controls in research setting (Jamaluddin et al., 2009[5]) and the use of dual baseline approach (which increase test retest stability coefficient or ‘ceiling effect’) as used by Duff et al. (2001[2]).

Another concern of practice-related measurement error is item-specific practice. This occurs when participants are asked to memorize the same items on more than one occasion. As mentioned above, the test-specific practice that is related to the familiarity of procedure and better strategies in the next memory test is difficult to detect and change. However, the item or content-specific practice of the test can be reduced by using alternate test forms (Benedict and Zgaljandic, 1998[1]). Most previous studies demonstrated that there is significant difference in the rate of improvement when the same or alternate forms of both verbal and nonverbal learning tasks are used repeatedly (Benedict and Zgaljandic, 1998[1]). Alternate form is more suitably administered when it is based on declarative memory/spatial processing compared to procedural/verbal fluency. 

The validated Malay version of Auditory Verbal Learning Test (MAVLT) is found to have good discriminatory validity when comparing healthy population with schizophrenia patients (Jamaluddin et al., 2009[5]). The MAVLT has been used in memory testing among smokers (Hamzah et al., 2012[4]), chronic renal failure patients undergoing haemodialysis (Othman et al., 2014[9]), and postmenopausal women receiving honey supplementation (Othman et al., 2011[10]). It is high time that alternate forms of MAVLT be developed so as to minimize item-specific practice effect during repeated neuropsychological evaluation. Therefore, the present study aimed to 

(i) develop alternate forms for MAVLT from original MAVLT i.e. Lezak (1983[7]) and Shapiro and Harrison (1990[13]), taking into account the linguistic criteria specific to Malay culture and language 

(ii) determine the equivalency and practice effect of the newly developed alternate forms.

## Materials and Methods

### Development of two alternate forms for MAVLT

The Alternate Form 1 was developed based on the earlier translated and validated MAVLT from Lezak (1983[7]) and the Alternate Form 2 was translated and modified from the original Shapiro and Harrison (1990[13]). For Alternate Form 1, the words in list A, B and recognition were selected from the 5 categories i.e. animal, vehicle, body parts, household objects and tools as in the MAVLT forms. For Alternate Form 2, of the 30 words from the 2 original English word lists, 24 words which were in accordance with the desired linguistic criteria were retained from both lists A and B. Six new words adapted to the Malay language were chosen for the development of the Malay lists. 

Finally, the word items on these newly formed Malay lists were compared with the original lists for consistency in terms of word length, and all were one- or two-syllable concrete nouns. There were no obvious semantic or phonetic associations or similarities between the words on the same list and they were chosen from amongst frequently occurring words in the Malay language. The probability of the occurrence of the word in common usage in the Malay language was ascertained using the Malay word count by Le Prevost (1952[6]). Recognition lists were constructed using target words and adding 20 new semantically associated or phonetically similar words as distracters. The above criteria helped to establish form equivalence between the two new lists. Item characteristics of the original MAVLT and new adapted Malay word lists in Alternate Form 1 and 2 were as shown in Table 1(Tab. 1).

### Participants

The study was carried out on 90 healthy volunteers; 19 males (21.1 %) and 71 (78.8 %) females. Their age range was between 20-29 years old with median age of 21 years old for all the groups. They were recruited from first and second year nursing students of School of Health Sciences, Health Campus, Universiti Sains Malaysia. The number of female participants was more than male participants (80:20) reflecting the gender distribution of students in the nursing program. 

Written informed consent was obtained from each participant before commencement of the study. Participants were chosen among nursing students for the following reasons; (i) the need for homogenous sample to reduce ceiling effect and (ii) the need for participants’ cooperation to reduce the dropout rate as this study procedure required each participant to complete 2 or 3 visits. Participants were not enrolled if they had history of brain surgery, psychiatric illness or any type of medical condition, or if they had history of using medication that might affect cognitive functioning.

### Study procedure and design

This study protocol was approved by the Research & Ethics Committee, Universiti Sains Malaysia. The study was carried out in Hospital Universiti Sains Malaysia (HUSM), Kubang Kerian, Kelantan. Participants were briefed on the nature of the study and informed consent was obtained at the initial visit. Randomization was computer-generated and participants were identified by their number in the students list and randomly assigned to one of three groups; Group 1 (MAVLT-MAVLT), Group 2 (MAVLT- Alternate Form 1- Alternate Form 1) and Group 3 (MAVLT- Alternate Form 2- Alternate Form 2). The test administration was standardized as described earlier by Lezak et al. (2004[7]). 

The first group was tested upon the MAVLT in two test sessions with intersession intervals of one month; the second group received MAVLT, Alternate Form 1, and Alternate Form 1, respectively, with one month intersession and the third group received MAVLT, Alternate Form 2, and Alternate Form 2, respectively, with one month intersession. The computer-assisted memory test was administered to the participants by the same interviewer who was trained by the second author (a psychiatrist) and was not involved in randomization and grouping of participants.

Each memory test consists of two different lists (A and B) of 15 concrete nouns. Participants were asked to listen to the first list (A) five times (A1 to A5) at a rate of one item per second (computer-assistance was used to standardize the rate). Free verbal recall (immediate memory) was tested immediately after each presentation. Total learning (A1+A2+A3+A4+A5) reflects the acquisition phase in the memory information processing operations. Then the participants were asked to listen to a second list (B) followed by its free recall. Thereafter, recall of list A (A6) was examined without prior presentation of list A. After 20 minutes of rest, recall of list A (A7) was repeated again without its prior presentation. Finally, participants had to recognize the words from list A interspersed among semantically or phonetically related words in a third list comprising of 30 words. 

### Statistical analysis

Descriptive analysis was done on mean score of each test among the visits. Repeated measure ANOVA within group analysis was performed followed by pair wise comparison with 95 % confidence interval adjustment by Bonferroni correction to demonstrate equivalency and practice effect.

## Results

### Mean scores of MAVLT at baseline and Alternate Form at an interval of one month

The mean score of trials A1 to A5 at baseline using MAVLT was not significantly different among the three groups; the mean score differences were noted to be at most of 2 points difference among each other which is still within the standard deviation. 

Trial A1-A5 represents the acquisition or learning phase in this test with A5 as maximum learning. For total score of A1-A5 which represents total learning, the mean score differences between groups were noted to be at most of 4 points difference among each other. Similar trend was also noted with the rest of the measurements such as A6 (post interference recall), A7 (delayed recall) and recognition list.

At month 1, the trend of mean score was similar across measures as well as at month 2. Thus it can be assumed that the descriptive analysis of mean score across trial in all forms at different times yield comparable result. The mean scores of each trial for the three groups are shown in Table 2(Tab. 2).

### Equivalency of Alternate Form 1 and Alternate Form 2 with MAVLT

Equivalency of Alternate Forms 1 and 2 with MAVLT was performed by comparing the mean score of each trial between MAVLT and either Alternate Form 1 or 2 at one month interval. There were no significant difference in the mean score of trials A1, A2, A3, A5, B, A7 and recognition list between MAVLT and Alternate Form 1 and the mean score of trials A1, A3, A4, A5, B, A6, A7 and recognition list between MAVLT and Alternate Form 2 (Table 3(Tab. 3)). 

### Practice effects of MAVLT and alternate forms

Practice effect was checked by comparing the mean score of each trial using the same form. There was significant improvement in the mean score at baseline and after one month as expected in group 1. Pair wise comparison with Bonferroni correction showed significant improvement in the mean scores of trial A1, A2, A3 and A1-A5. However there was no significant difference in the mean score of trial A4, A5, A6, A7 and recognition (Table 4(Tab. 4)). 

For group 2, when compared between the scores at 1 month and at 2 months using the same Alternate Form 1, there were significant improvement in the mean scores of most of thetrials A1, A2, A4, A5 and A1-A5 except trials A3, A6, A7 and recognition (Table 4(Tab. 4)). When compared between the scores at 1 month and at 2 months using the same 

Alternate Form 2 for group 3, the results were comparable to the practice effects using MAVLT whereby significant improvement were observed in the mean scores of trials A1, A2, A3 and A1-A5 (Table 3(Tab. 3)).

## Discussion

Vakil and colleagues (2004[14]) reported that age, intelligence and population type will affect the score in AVLT. This study was conducted in a homogenous population similar to the earlier study (Shapiro and Harrison, 1990[13]). The population of nursing students was chosen in the present study to reduce the variability in terms of age, gender, estimates on intelligence and mental status. The study by Shapiro and Harrison (1990[13]) used university students in Virginia as sample in which 25 students with mean age of 19 were recruited. However, later studies by Geffen et al. (1994[3]) and Rezvanfard et al. (2011[11]) sampled from the general population i.e. volunteers in Australia and Iran respectively, and their results may represent that of a more heterogeneous population. 

In the present study, repeated measurement of parallel design was chosen, almost similar to the study by Rezvanfard et al. (2011[11]) with fixed sequence (non counterbalanced design). Studies with multiple alternate forms otherwise prefer to use counterbalanced method such as in other studies (Shapiro and Harrison, 1990[13]; Geffen et al., 1994[3]). The counterbalance design has advantage on order effect of test, which was not tested in the present study and should be looked into in future study. 

The descriptive statistics of the present study were comparable to other studies. Rezvanfard and colleagues (2011[11]) showed almost similar mean scores in lists 1, 2 and 3. In addition, Geffen and colleagues (1994[3]) described similar scores in their study in both forms tested in all the trials. 

There was no significant difference in the mean scores of all the trials when comparing between MAVLT and Alternate Form 2 except for trial A2. The Alternate Form 1, however, showed significant difference in the mean scores in trials A4, A6 and A1-A5. These results suggest that Alternate Form 2 is more equivalence to the original form (MAVLT) compared to Alternate Form 1. This could be due to the word selection procedures of Alternate Form 1 which was similar to the original UCLA and MAVLT forms and the participants developed better test taking strategies.

The present study showed that utilization of the same list or same word selection procedure yield significant improvement in the mean scores i.e. larger practice effect in re-administration. However, use of alternate form reduced the practice effect as shown by no improvement in the mean score between baseline and month 1 especially when using Alternate Form 2. Study of Benedict and Zgaljardic (1998[1]) used multiple alternate forms and found improvement in score even with alternate form when tested on more than two sessions. The participants were also more likely to develop better test taking strategies rather than depending on the item itself. Shapiro and Harrison (1990[13]) discuss that the general practice effect in repeated neuropsychological testing will remain even when using alternate form which is only capable of eliminating the item-specific practice effect.

In the present study, the study interval was one month and this is similar to an earlier study by Rezvanfard et al. (2011[11]). The shortest interval used by Ryan and Geisser (1986) where the study yielded high reliability was 90 minutes to 2 hours. Better reliability was seen in a study by Shapiro and Harrison (1990[13]). They used a study interval of 2 days to 13 days. When the interval of re-testing was increased, the reliability coefficient becomes lower as observed by Rezvanfard and colleagues (2011[11]). They also found that repeated administration of the alternate form after one month could remove the undesirable practice effects as observed in the present study. Other factors such as the motivation of the participants as well as the circumstances or environment where the test was conducted may affect the results.

In conclusion, the MAVLT is a reliable instrument for repeated neuropsychological testing as long as alternate forms are used. The Alternate Form 2 showed better equivalency to MAVLT and produced less practice effects when used as an alternate form for repeated neuropsychological testing. 

## Acknowledgements

This research was funded by a Universiti Sains Malaysia short-term grant (PPSP/61313056). The authors also thank the Dean and nursing students of School of Health Sciences, Universiti Sains Malaysia who participated in this study.

## Figures and Tables

**Table 1:**
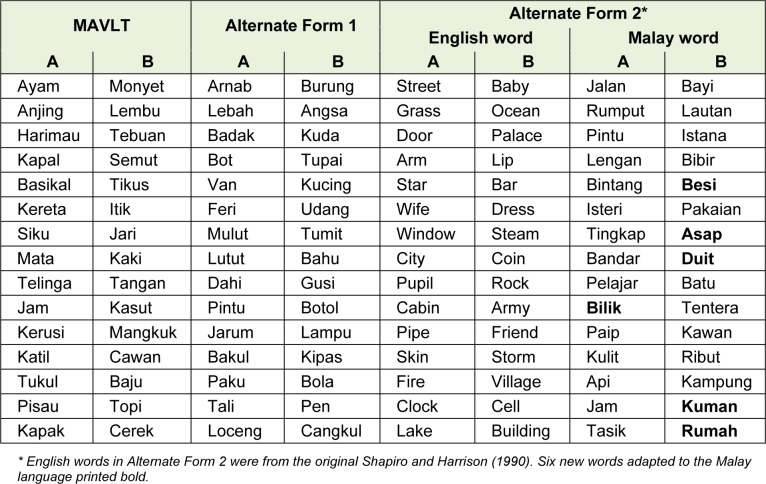
Item characteristics of the original MAVLT and new adapted Malay word lists in Alternate Form 1 and 2

**Table 2:**
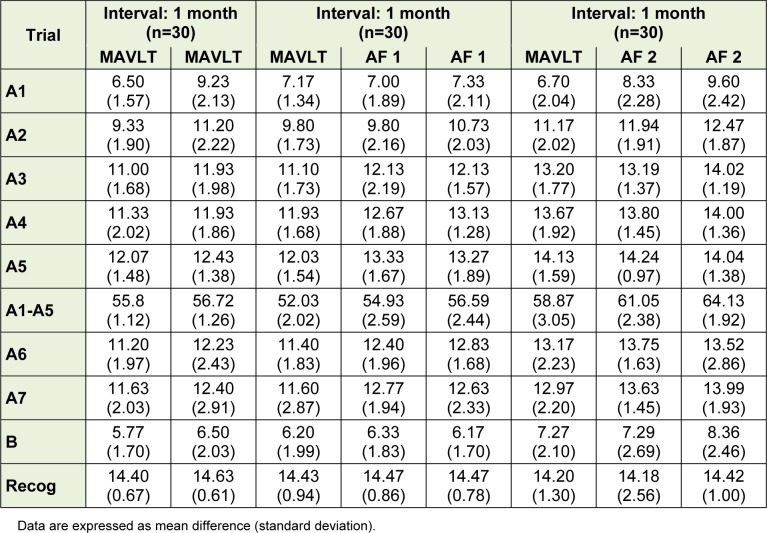
Descriptive statistics for the mean scores of MAVLT at baseline and alternate form at an interval of one month

**Table 3:**
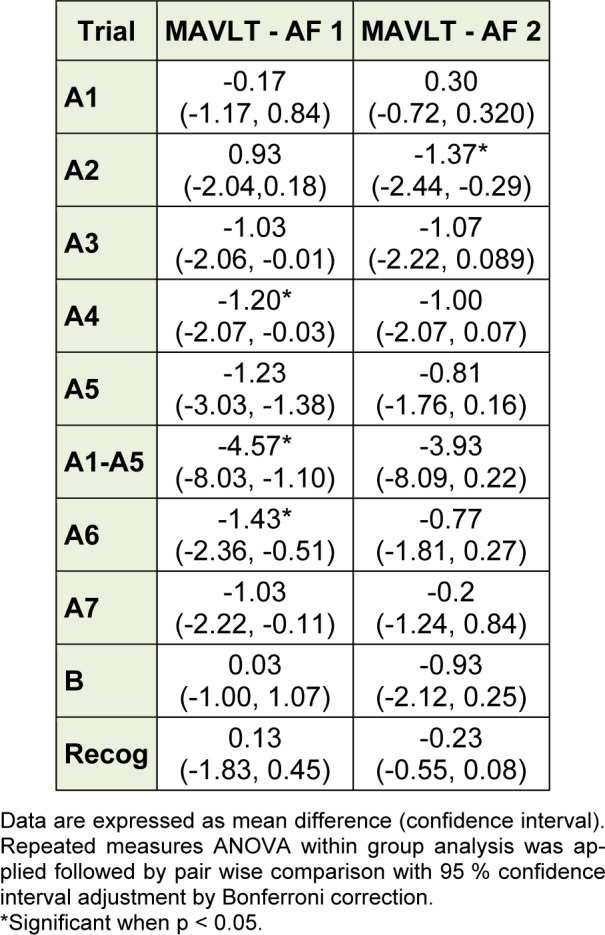
Equivalency of Alternate Form 1 and Alternate Form 2 with MAVLT

**Table 4:**
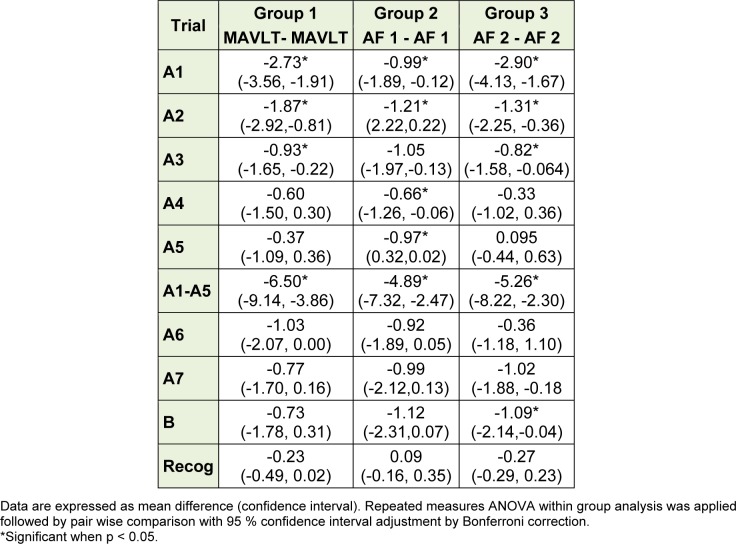
Practice effects of MAVLT and alternate forms
